# Modeling factors influencing the demand for emergency department services in ontario: a comparison of methods

**DOI:** 10.1186/1471-227X-11-13

**Published:** 2011-08-19

**Authors:** Rahim Moineddin, Christopher Meaney, Mohammad Agha, Brandon Zagorski, Richard Henry Glazier

**Affiliations:** 1 Department of Family and Community Medicine, University of Toronto, Ontario, Canada; 2 Institute for Clinical Evaluative Sciences, Toronto, Ontario, Canada; 3 Dalla Lana School of Public Health, University of Toronto, Ontario, Canada; 4 Centre for Research on Inner City Health, The Keenan Research Centre in the Li Ka Shing Knowledge Institute of St. Michael's Hospital, Ontario, Canada; 5 Department of Health Policy, Management and Evaluation, University of Toronto, Ontario, Canada; 6 Pediatric Oncology Group of Ontario, Toronto, Ontario, Canada

## Abstract

**Background:**

Emergency departments are medical treatment facilities, designed to provide episodic care to patients suffering from acute injuries and illnesses as well as patients who are experiencing sporadic flare-ups of underlying chronic medical conditions which require immediate attention. Supply and demand for emergency department services varies across geographic regions and time. Some persons do not rely on the service at all whereas; others use the service on repeated occasions. Issues regarding increased wait times for services and crowding illustrate the need to investigate which factors are associated with increased frequency of emergency department utilization. The evidence from this study can help inform policy makers on the appropriate mix of supply and demand targeted health care policies necessary to ensure that patients receive appropriate health care delivery in an efficient and cost-effective manner. The purpose of this report is to assess those factors resulting in increased demand for emergency department services in Ontario. We assess how utilization rates vary according to the severity of patient presentation in the emergency department. We are specifically interested in the impact that access to primary care physicians has on the demand for emergency department services. Additionally, we wish to investigate these trends using a series of novel regression models for count outcomes which have yet to be employed in the domain of emergency medical research.

**Methods:**

Data regarding the frequency of emergency department visits for the respondents of Canadian Community Health Survey (CCHS) during our study interval (2003-2005) are obtained from the National Ambulatory Care Reporting System (NACRS). Patients' emergency department utilizations were linked with information from the Canadian Community Health Survey (CCHS) which provides individual level medical, socio-demographic, psychological and behavioral information for investigating predictors of increased emergency department utilization. Six different multiple regression models for count data were fitted to assess the influence of predictors on demand for emergency department services, including: Poisson, Negative Binomial, Zero-Inflated Poisson, Zero-Inflated Negative Binomial, Hurdle Poisson, and Hurdle Negative Binomial. Comparison of competing models was assessed by the Vuong test statistic.

**Results:**

The CCHS cycle 2.1 respondents were a roughly equal mix of males (50.4%) and females (49.6%). The majority (86.2%) were young-middle aged adults between the ages of 20-64, living in predominantly urban environments (85.9%), with mid-high household incomes (92.2%) and well-educated, receiving at least a high-school diploma (84.1%). Many participants reported no chronic disease (51.9%), fell into a small number (0-5) of ambulatory diagnostic groups (62.3%), and perceived their health status as good/excellent (88.1%); however, were projected to have high Resource Utilization Band levels of health resource utilization (68.2%). These factors were largely stable for CCHS cycle 3.1 respondents. Factors influencing demand for emergency department services varied according to the severity of triage scores at initial presentation. For example, although a non-significant predictor of the odds of emergency department utilization in high severity cases, access to a primary care physician was a statistically significant predictor of the likelihood of emergency department utilization (OR: 0.69; 95% CI OR: 0.63-0.75) and the rate of emergency department utilization (RR: 0.57; 95% CI RR: 0.50-0.66) in low severity cases.

**Conclusion:**

Using a theoretically appropriate hurdle negative binomial regression model this unique study illustrates that access to a primary care physician is an important predictor of both the odds and rate of emergency department utilization in Ontario. Restructuring primary care services, with aims of increasing access to undersupplied populations may result in decreased emergency department utilization rates by approximately 43% for low severity triage level cases.

## Background

Emergency departments are medical treatment facilities, designed to provide episodic care to patients suffering from acute injuries and illnesses as well as patients who are experiencing sporadic flare-ups of underlying chronic medical conditions which require urgent medical attention [1]. The scientific literature suggests that demand for emergency department services has been increasing over recent decades in many geographic jurisdictions, including: Singapore [2], Spain [3] and the United States [4]. Changing preferences of medical consumers may be related to this increased demand for emergency health services. For example, research suggests that certain sub-groups of patients may not have access to a primary care provider at all, and use the emergency department as a regular source of care [5]. For those who can access primary care in the community, their choice to visit an emergency department may be attributable to the convenience and ease of access to emergency services, relative to primary care services, in their geographic locations [3,6]. In other jurisdictions, it has been observed that a small proportion of patients account for a relatively large utilization of emergency services. These individuals have been coined "heavy users", "repeaters" or "frequent flyers" [7,8]. Qualitative studies have shown that these heavy users are typically characterized by a high prevalence of psycho-social limitations and associated medical co-morbidities. The complex nature of the diseases which afflict these patients makes them difficult to treat via emergency medicine, and many are better treated via multi-facetted and individually tailored treatment plans in the community [9,10]. In Ontario, per capita demand for emergency department services decreased 10% between 1993 and 2000; however, concurrent hospital closures over this time period resulted in surviving facilities experiencing increased patient volumes [1].

Increased patient volumes at emergency departments, resulting from changes in patient preference/demand characteristics, decreasing supply of emergency department resources (eg. treatment facilities, physicians, nurses), or long term structural changes to patient case mix as a result of demographic trends have resulted in documented challenges in the delivery of emergency department services. These challenges include: increasingly long wait times, ambulance diversions, and crowding. Despite considerable research in this area, a lack of consensus exists as to the most appropriate strategies for addressing these problems. A review of available literature can sometimes illustrate contradictory findings regarding the characteristics of those individuals whom exhibit increased (sometimes coined "inappropriate") demand for emergency department services. One area of controversy is whether lack of access to a primary care physician in the community is attributable to increased utilization of emergency department services. In an Ontario based study, Chan [1] found that the majority of repeat emergency department users also have periodic contact with primary care physicians. This is a similar finding to that of Andren [11] who did not observe a difference in utilization of primary care physicians between repeat users or non-repeat users of emergency department services. Conversely, studies from Quebec [12] and Brazil [13] point to lack of access to community based physicians and poor continuity of care as being chief predictors of emergency department demand in their respective samples. Another, interesting predictor of emergency department utilization is the patient's location of primary residence. Studies from Ontario [6] and Quebec [12] suggest that patients with rural residences use emergency department services at greater rates than non-rural residences. While the assessment of these factors are two of the main objectives of this paper, we will also explore other possible causes for increased demand for emergency department services, including: age, gender, education, income, perceived health status and comorbidity status. All of these data are collected at a patient level, and as such, inferences from this study are not subject to issues regarding ecological fallacy, a distinguishing feature from previous population studies in emergency medicine. We will also stratify these analyses by the severity of an individual's triage score at time of presentation to the emergency department. This will allow us to assess whether factors influencing demand are the same in high severity cases as they are in lower severity cases.

Another distinguishing feature of this study is the methodology employed to assess the factors' contributing to demand for emergency department services. Previous studies have either dichotomized the count of emergency department visits at some threshold (indicating non-frequent users versus frequent users) and modeled the transformed outcome using logistic regression [6,11] whereas, other studies have modeled the count outcome using Poisson regression [12]. The former strategy may not be ideal because categorization results in some loss of information. The latter strategy may not be appropriate because the Poisson model is not capable of accounting for the heteroskedasticity, unobserved heterogeneity and the large frequency of zero counts that occur when patients in a population based study do not visit the emergency department over a given period of time. A more amenable analytic approach would be to use a less restrictive model that does not assume that the conditional variance of the response is equal to the conditional mean - such as the negative binomial regression model. Novel regression methods such as the zero-inflated Poisson (ZIP), zero-inflated negative binomial (ZINB), hurdle Poisson (HP) and hurdle negative binomial (HNB) models have also been considered in the fields of economics [14,15], traffic accident research [16], childhood development [17], food microbiology [18] and pharmaceutical research [19] for modeling count data which contain an excess of zero count observations. In this paper, we fit all 6 regression models (Poisson, Negative Binomial, ZIP, ZINB, HP and HNB) and compare them to assess the most appropriate model for this sample of data. Once we have established an appropriately fit model we interpret the estimated coefficients in an attempt to enhance our understanding about the factors influencing demand for emergency department services in Ontario.

## Methods

### Data Sources and Study Population

The Canadian Community Health Survey (CCHS) cycles 1.1 to 5.1 are national surveys which have been conducted by Statistics Canada from 2000 to 2010 [20]. The CCHS is designed to provide timely cross-sectional estimates of health determinants, health status and health system utilization at a sub-provincial level (health region or combination of health regions). The target population of the CCHS includes household residents in all provinces and territories, with the exception of individuals in First Nations reserves, Canadian Armed Forces Bases and some remote areas. The CCHS employs a multi-stage stratified cluster design and the Ontario portion of the survey consisted of more than 25,000 respondents in each cycle.

In the province of Ontario CCHS respondents were asked to provide their Ontario health card numbers and to consent to linkage of their CCHS responses with personal health care utilization data. Those consenting in cycles 1.1-3.1 were linked to the Ontario Registered Persons Database (RPDB), the province's health care registry. Once linked with the RPDB, health card numbers were used to link respondents with fee-for-service billing claims that physicians report to the Ontario Health Insurance Plan (OHIP). These data were collected for 2000-2001 and 2002-2003 observation periods. Approximately 94% of all physician encounters in the province are included in this database. A small number of physicians are salaried employees and hence do not directly bill OHIP for patient encounters. Records of all emergency department visits were also submitted to the Canadian Institute for Health Information (CIHI) as part of the National Ambulatory Care Reporting System (NACRS), for which close to 100% of emergency department claims in the province are included. The data were accessed at the Institute for Clinical Evaluative Sciences (ICES) as part of a comprehensive research agreement with the Ontario Ministry of Health and Long-Term Care (MOHLTC).

The study setting of Ontario is Canada's most populous province and the second largest province in terms of geographic area. The study population was restricted to individuals between the ages of 20 and 79 years to avoid proxy responses that could be assigned to children and older seniors. The cycles 2.1 collection period was January 2003 through December 2003 and cycle 3.1 was January 2005 through December 2005.

### Outcome Variables

The number of emergency department visits during the 365 day interval following the interview date were tallied for fiscal years 2003 through 2006 for each individual respondent of CCHS cycle 2.1 and 3.1, and counted using the NACRS database. The scrambled Ontario health card number was used as a unique key to link individual level medical, socio-demographic, psychological and behavioral data from the CCHS 2.1 and 3.1 to emergency department visit data. We defined a potentially avoidable emergency department visit as one with a Canadian Triage and Acuity Scale (CTAS) score of 4 or 5 (less urgent), where the patient was not admitted to the hospital following observation by the physician. We defined an unavoidable emergency department visit, as one with a CTAS score of 1, 2 or 3 (urgent) and no diagnostic code indicating an injury. We assume these emergency department visits are unlikely treatable in a primary care environment. We excluded emergency department visits where the patient left without being seen and excluded transfers (i.e., kept the first emergency department visit when there was a transfer) and pregnant women. Outcome variables for each participant are the number of less urgent and the number of urgent emergency department visits. In regression models, participants with no emergency department visits were included with zero visits for both less urgent and urgent emergency department visits.

### Assessment of Comorbidity

We used the John Hopkins University Ambulatory Care Groups Case Mix Adjustment System (version 7) to summarize the degree of comorbidity experienced by Ontarians during the two year period prior to the interview date. This software reads in international classification of disease (ICD) codes from physician and hospital-based claims and categorizes patients as having up to 32 different Ambulatory Diagnostic Group (ADG) labels. An individual can be assigned to multiple ADG's depending on their respective diagnoses. We collapsed the 32-category variable into three categories, specifically: individuals falling into 0-5, 6-9 and greater than 9 ADG's. This three level categorical variable was used as an indicator of comorbidity in all subsequent analyses.

The Ambulatory Care Groups Case Mix Adjustment software also generates Resource Utilization Bands (RUB), which estimate expected resource utilization. Patient level RUB categorization is determined through consideration of age, sex, and disease diagnoses. Different categories of RUB are associated with different levels of expected resource use and overall cost to the health care system over a given period of time. RUB values vary from 0-5, with higher values associated with higher utilization levels. For this study, RUBs were categorized as ≥ 4 (very high), 3 (high), 2 (medium), and 0-1 (low). The ACG measures were determined using two years of diagnostic data (fiscal year 2003 and 2004) from physician and hospital-based claims.

### Predictors

Individual-level variables that were included in the regression models were gender (male, female), age (20-44, 45-64, 65-79), total household income (low {less than $20,000}, medium {$20,000-$59,999}, high {more than $60,000}), education (low {not completed high school}, medium {high school completion and some post-secondary education} and high {university degree}), number of chronic medical conditions (0, 1, >1) from the following list (asthma, fibromyalgia, arthritis/rheumatism, back problems, high blood pressure, diabetes, epilepsy, heart disease, and cancer), perceived health status (poor/fair, good, very-good/excellent), number of ADG's (0-5, 6-9, >9), RUB status (0-1, 2, 3, 4-5), access to a primary care physician in the community (no, yes), and location of primary residence (rural, urban).

### Analytic Methods

Population studies which seek to estimate demand for emergency department services or hospitalization typically exhibit a large proportion of zeroes, representing the persons that do not use any of the services being investigated during the observational period of interest. Factors influencing the demand for these services are routinely modeled using Poisson or negative binomial regression. While the negative binomial regression model does not impose as stringent a set of restrictions on the conditional mean-variance relationship as the Poisson model, neither is ideal for handling data with a large proportion of zeroes. Failure to account for the mass of zeros that are occurring at a greater proportion than would be predicted by either the Poisson or negative binomial models may result in biased parameter estimates and misleading inferences. Several methods are proposed for analyzing data with excess zeros, including: the ZIP, ZINB, HP and HNB regression models. Given the lack of use of these models in emergency medical research we will describe each method below.

Before proceeding to any multiple regression modeling, descriptive statistics were generated to characterize the sample under investigation. For continuously distributed variables we presented means and standard deviations; whereas, for categorical variables we presented counts and percentages.

### Regression Models for Count Outcomes

Perhaps the most parsimonious and widely implemented method for modeling count data in the public health sciences is Poisson regression. The Poisson regression model assumes that the number of events (y_i_) experienced by patient i follows a Poisson distribution:

$$P\left(Y_{i}=y_{i}|x_{i}\right)=\frac{e^{-\mu_{i}}\mu_{i}^{y_{i}}}{y_{i}!}$$

where μ_i _represents the conditional mean response of a given patient, which is assumed to depend on a set of observed data (x_i_) and an estimated vector of coefficients (β). Mathematically, this relationship takes the following form:

$$E\left(y_{i}|x_{i}\right)=\mu_{i}=e^{x_{i}\beta }$$

Taking the natural logarithm of the conditional mean allows for the response under consideration to vary linearly as a function of observed predictor variables multiplied by the effect of their corresponding regression coefficients. Various numerical maximization methods exist for iteratively estimating the values of the coefficient vector, β, and the associated -covariance matrix. variance Estimates are typically found by finding the parameter estimates that maximize the following log-likelihood function:

$$LL_{Poisson}=\overset{n}{\underset{i=1}{\sum }}\left[-\mu_{i}+y_{i}ln\left(\mu_{i}\right)-ln\left(y_{i}!\right)\right]$$

Since the natural logarithm of the likelihood function for the Poisson regression model is globally concave, a unique maximum can be found if it exists [21]. A restrictive assumption attached to the Poisson regression model is that the conditional variance is assumed to be equal to the conditional mean. As a result, the Poisson regression model is not always an ideal model for count data, especially in instances where a large mass of observations exists on the corner of the empirical distribution. This typically arises in the form of observed zeroes in a data set that are in excess of what would be predicted by the Poisson distribution. In severe instances, fitting a Poisson model to data with excess zeroes can result in model misspecification, inefficient parameters estimates and incorrect inferences.

A less parsimonious, but more flexible extension to the Poisson regression model is the negative binomial regression model. The negative binomial regression model does not assume that the conditional variance of the response is equal to the conditional mean. A simple extension to the specification of the Poisson conditional mean leads to a negative binomial regression model, which is illustrated below:

$$E\left(y_{i}|x_{i}\right)=\mu_{i}=e^{x_{i}\beta +\varepsilon_{i}}=e^{x_{i}\beta }e^{\varepsilon_{i}}=e^{x_{i}\beta }\delta_{i}$$

Above, the conditional mean for the Poisson model has been adjusted by adding an individual specific random term, ε_i_, that is assumed to be uncorrelated with the observation vector, x_i_. Typically one assumes that $\delta_{\text{i}}=e^{\varepsilon_{i}}$ follows a gamma distribution. By extending the Poisson conditional mean in this manner, we arrive at the negative binomial regression model. The inclusion of the random error in the conditional mean of the negative binomial regression model is useful, as it allows for the modeling of both observed and unobserved heterogeneity whereas, the Poisson model only accounts for observed heterogeneity. In other words, using the Poisson regression model it was assumed that patients with the same observation vector would incur the same conditional mean response. The incorporation of the random term in the negative binomial regression model allows patients with identical observation vectors to experience different conditional mean responses. If we assume (ε_i_) has a mean that of 1 and variance of υ then the conditional mean of yi is still μ_i_; however, the conditional variance becomes μ_i_(1 +uμ_i_) = μ_i _+ uμ_i_^2^. As u approaches zero binomial regression model converges toward the Poisson model, with a conditional mean that is equal to the conditional variance, μ_i _[19,21]. For the negative binomial model, the probability that an individual patient incurs yi emergency department visits is dictated by the following density function:

$$P\left(Y_{i}=y_{i}|x_{i\prime }v\right)=\frac{\Gamma \left(y_{i}+\frac{1}{v}\right)}{\Gamma \left(y_{i}+1\right)\Gamma \left(\frac{1}{v}\right)}{\left[\frac{\frac{1}{v}}{\frac{1}{v}+\mu_{i}}\right]}^{\frac{1}{v}}{\left[\frac{\mu_{i}}{\frac{1}{v}+\mu_{i}}\right]}^{y_{i}}$$

Above, μ_i _represents the mean number of events that is expected for an individual with observation vector xi, u represents the negative binomial dispersion parameter and Γ(·) represents the gamma function. Determination of regression coefficients in negative binomial regression proceeds by maximizing the following log-likelihood function with respect to the unknown parameters:

LLNB= ∑i=1nlnΓ(yi+1v)Γ(yi+1)Γ1v-yi+1vln(1+vμi)+yi ln(vμi)

The negative binomial regression model is a useful model for accounting for data in which unobserved heterogeneity or temporal/spatial correlation is present; however, it is not necessarily an optimal model for dealing with data that contain an excess mass of zeroes at the corner of its empirical distribution.

Zero Inflated Poisson (ZIP) regression models were introduced by Lambert [22] as a method for modeling the factors influencing the number of defects encountered in a manufacturing application. Greene [23] introduced the idea of the Zero Inflated Negative Binomial (ZINB) model to handle both excess zeroes and over-dispersion as a result of unobserved heterogeneity which commonly arises in economic problems. Each of the models - ZIP and ZINB - assumes that patients can fall into one of two groups. The first group of patients never experience the outcome (eg. always show zero demand for emergency department services) and the second group of patients show some positive demand which is governed by the Poisson or negative binomial density. A patient falls into group 1 with probability ψ_i_, and a patient falls into group 2 with probability (1 - ψ_i_), where ψ_i _is an estimable parameter from available data. Group 1, although homogeneously comprised of persons with zero demand for emergency services over a given interval of time are derived from a combination of processes - resulting from the binomial probability, ψ_i_, and the zeroes which accumulate naturally in the Poisson or negative binomial densities. Distinguishing between the two sources of zeroes is not possible, as it is a form of discrete unobserved heterogeneity [21]. The probability density function for the ZIP model is given below:

$$P\left(Y_{i}=y_{i}|x_{i\prime }\psi_{i}\right)=\left\{\begin{array}{cc} \psi_{i}+\left(1-\psi_{i}\right)e^{-\mu_{i}}\; & y_{i}=0\\ \left(1-\psi_{i}\right)\frac{e^{-\mu_{i}}\mu_{i}^{y_{i}}}{y_{i}!} & y_{i}>0 \end{array}\right)$$

Similarly, for zero-inflated negative binomial model, the probability density function is given by:

$$P\left(Y_{i}=y_{i}|x_{i\prime }\psi_{i\prime }v\right)=\left\{\begin{array}{cc} \psi_{i}+\left(1-\psi_{i}\right)\frac{1}{{\left(1+v\mu_{i}\right)}^{1∕v}}\; & y_{i}=0\\ \left(1-\psi_{i}\right)\frac{\Gamma \left(y_{i}+\frac{1}{v}\right)}{\Gamma \left(y_{i}+1\right)\Gamma \left(\frac{1}{v}\right)}\frac{{\left(v\mu_{i}\right)}^{y_{i}}}{{\left(1+v\mu_{i}\right)}^{y_{i}+\frac{1}{v}}}\; & y_{i}>0 \end{array}\right)$$

For both the ZIP and ZINB models the probability of an excess zero, ψ_i_, the is modeled using logistic regression (although, any binary regression framework will suffice). As a result, the probability of an excess zero is given by:

$$\psi_{i}=\frac{1}{1+e^{\eta_{i}}}=\frac{1}{1+e^{z_{i}\gamma }}$$

In other words, the probability of an excess zero is a function of some observed linear predictor, η_i_, which itself is formed from a set of predictor variables, z_i_, multiplied by their associated logistic regression coefficients, ε(nb. the set z_i_, in the logistic of model need not equal the set of variables, x_i_, in the Poisson or negative binomial component regression models). For the ZIP model the conditional mean and variance are:

$$\begin{array}{c} E\left(y_{i}|x_{i\prime }z_{i}\right)=\mu_{i}-\mu_{i}\psi_{i}\\ Var\left(y_{i}|x_{i\prime }z_{i}\right)=\mu_{i}\left(1-\psi_{i}\right)\left(1+\mu_{i}\psi_{i}\right) \end{array}$$

For the ZINB model, the conditional mean is the same as for the ZIP model; however, the conditional variance differs. The equations for both the conditional mean and variance of the ZINB model are given below:

$$\begin{array}{c} E\left(y_{i}|x_{i\prime }z_{i}\right)=\mu_{i}-\mu_{i}\psi_{i}\\ Var\left(y_{i}|x_{i\prime }z_{i}\right)=\mu_{i}\left(1-\psi_{i}\right)\left(1+\mu_{i}\left(\psi_{i}+v\right)\right) \end{array}$$

Considering ψ_i _as the probability of excess zeroes, it can be observed that as ψ_i _tends toward zero then the probability densities, as well as the conditional mean and variances of the ZIP and ZINB models converge toward the corresponding formulas for the Poisson and negative binomial models, respectively [18,19,21]. Determination of regression coefficients for the ZIP and ZINB models once again occurs by maximization of the log-likelihood functions, which are given below.

$$LL_{ZIP}=\sum_{i=1}^{n}\left[I(y_{i}=0)\ln[(\psi_{i}+(1-\psi_{i})\exp(-\mu_{i})]+I(y_{i}\geq 1)[\ln(1-\psi_{i})+y_{i}\ln(\mu_{i})-\mu_{i}-\ln(y_{i}!)]\right]$$

$$\begin{array}{c} LL_{ZINB}=\sum_{i=1}^{n}[I(y_{i}=0)\ln(\psi_{i}+(1-\psi_{i})\frac{1}{{\left(1+v\mu_{i}\right)}^{\frac{1}{v}}})+\\ I(y_{i}\geq 1)[\ln(1-\psi_{i})+\ln\left[\Gamma \left(y_{i}+\frac{1}{v}\right)\right]-\ln[\Gamma (y_{i}+1)]-\ln\left[\Gamma \left(\frac{1}{v}\right)\right]+y_{i}\ln(v\mu_{i})-(y_{i}+1/v)\ln(1+v\mu_{i})] \end{array}$$

Here *I*(·) is an indicator function.

One issue with the application of zero-inflated modeling strategies for emergency department demand is that interpretively some of the zeroes in ZIP/ZINB models are considered to be structural; whereas, others are assumed to arise as a result of a sampling process. Conceptually, it is hard to imagine even the healthiest individuals in the Ontario population not being "at risk" for an emergency department visit and hence representing a structural zero. As a result, even though the ZIP and ZINB models may fit our data well, a more parsimonious explanation of the phenomena under investigation can be derived using a hurdle modeling approach.

Hurdle models account for excess zeroes but are specified and interpreted slightly differently than the ZIP and ZINB models discussed above. The hurdle regression approach was introduced by Mullahy [14] and incorporates a Bernoulli or right censored count density (the hurdle component) with a left truncated count density (the count component). As a result, hurdle models are composed of two mechanisms, similar to their zero-inflated counterparts; however, hurdle approaches avoid modeling the probability of a zero occurrence as a function of two mixed sources - the structural component (logistic model) and a sampling component (count model). Rather, using a hurdle approach the probability of an observation occurring at zero or not zero is governed by a Bernoulli (or right censored count) density. That is, the probability of a zero occurrence is given by ψ_i_. Further, the probability of a non-zero occurrence is given by (1 - ψ_i_). Therefore, with probability (1 - ψ_i_) we will observe a positive integer count from either a truncated Poisson or negative binomial density (normalized to integrate to 1). In a mathematically more precise manner, Mullahy [14] illustrated that the general hurdle density is given as:

$$P\left(Y_{i}=y_{i}|x_{i},z_{i},\beta ,\gamma \right)=\left\{\begin{array}{ccc} & f_{zero}\left(0;\;z_{i},\gamma \right)\; & y_{i}=0\\ & \left(1-f_{zero}\left(0;z_{i},\gamma \right)\right)\frac{f_{count}\left(y_{i};x_{i},\beta \right)}{\left(1-f_{count}\left(0;x_{i},\beta \right)\right)}\; & y_{i}>0 \end{array}\right)$$

Above, z_i _and γ represent the respective variables and coefficients associated with the zero/non-zero (hurdle) process. The xi and β are the respective variables and coefficients associated with the count process. Lastly, the *f*_zero_(·) function represents either a Bernoulli or right censored count density; whereas, the *f*_count_(·) function typically represents a left truncated Poisson or negative binomial density.

If we purport to use a Bernoulli density to model the probability (ψ_i_) of a zero versus a non-zero (1 - ψ_i_) count, coupled with a left truncated Poisson density (with mean μ_i_) for the count process, then our overall hurdle Poisson (HP) density looks as follows:

$$P\left(Y_{i}=y_{i}|\psi_{i},\mu_{i}\right)=\left\{\begin{array}{cc} \psi_{i}\; & y_{i}=0\\ \left(1-\psi_{i}\right)\frac{1}{\left(1-e^{-\mu_{i}}\right)}\frac{e^{-\mu_{i}}\mu_{i}^{y_{i}}}{y_{i}!}\; & y_{i}>0 \end{array}\right)$$

Similarly, if we purport to use a Bernoulli density to model the probability (ψ_i_) of a zero versus a non-zero (1 - ψ_i_) count, coupled with a left truncated negative binomial density (with mean, μ_i_, and negative binomial dispersion parameter, υ) for the count process, then our overall hurdle negative binomial (HNB) density looks as follows:

$$P\left(Y_{i}=y_{i}|\psi_{i\prime }\mu_{i\prime }v\right)=\left\{\begin{array}{cc} \psi_{i}\; & y_{i}=0\\ \left(1-\psi_{i}\right){\left(1-\frac{1}{{\left(1+v\mu_{i}\right)}^{\frac{1}{v}}}\right)}^{-1}\frac{\Gamma \left(y_{i}+\frac{1}{v}\right)}{\Gamma \left(y_{i}+1\right)\Gamma \left(\frac{1}{v}\right)}\frac{{\left(v\mu_{i}\right)}^{y_{i}}}{{\left(1+v\mu_{i}\right)}^{y_{i}+\frac{1}{v}}}\; & y_{i}>0 \end{array}\right)$$

Again, regression coefficients are estimated through determination of the coefficients which maximize the following log-likelihood functions [18]:

$$LL_{HP}=\sum_{i=1}^{n}\left[I(y_{i}=0)\psi_{i}+I(y_{i}\geq 1)[\ln(1-\psi_{i})-\mu_{i}+y_{i}\ln(\mu_{i})-\ln(1-\exp(-\mu_{i})-\ln(y_{i}!)]\right]$$

$$\begin{array}{c} LL_{HNB}=\sum_{i=1}^{n}[I(y_{i}=0)\psi_{i}+I(y_{i}\geq 1)[\ln(\text{1}-\psi_{i})+\ln\left[\Gamma \left(y_{i}+\frac{1}{v}\right)\right]-\ln\left[{\left(\text{1}-\frac{\text{1}}{{\left(1+v\mu_{i}\right)}^{\frac{1}{v}}}\right)}^{-\text{1}}\right]-\\ \text{ln}[\Gamma (y_{i}+1)]-\ln\left[\Gamma \left(\frac{\text{1}}{v}\right)\right]-\left(y_{i}+\frac{\text{1}}{v}\right)\ln(1+v\mu_{i})+y_{i}\ln(v\mu_{i})]] \end{array}$$

Intuitively, the hurdle approach to handling excess zeroes in medical utilization data is appealing as predicted zeroes are not interpreted as a mix of structural and sampling zeroes. Rather, the first component of the hurdle approach can be used to model whether a person does or does not decide to seek emergency services over the time interval of our study. This process can be modeled using a binary regression framework, such as logistic or probit regression. Given that a person does decide to seek emergency services, the number of visits they make to the emergency department can then be modeled using a left truncated Poisson or negative binomial distribution.

### Comparing Regression Models for Count Outcomes

Vuong [24] proposed a likelihood ratio testing framework for non-nested model comparison and selection. To define the test, we begin by assuming there are two models, where $\hat{P_{1}}\left(y_{i}|x_{i}\right)$ is the probability of observing yi based on the first model and $\hat{P_{2}}\left(y_{i}|x_{i}\right)$ is the of observing yi based on the second model. If we further define

$$m_{i}=ln\;\left[\frac{\hat{P_{1}}\left(y_{i}|x_{i}\right)}{\hat{P_{2}}\left(y_{i}|x_{i}\right)}\right]$$

And let $\bar{m}$ represent the mean of the *m_i _*and the let s*_m _*represent the standard deviation of the *m_i_*. Then the Vuong statistic takes the following form:

$$V=\frac{\sqrt{N}\bar{m}}{s_{m}}$$

The Vuong statistic is asymptotically distributed as a N(0,1) variable. Calculating a normal based random confidence interval can be used to assess whether model 2 is favored over model 1, whether model 1 is favored over model 2, or whether insufficient evidence exists to claim either model is favored over the other [21]. Mathematically, if we let C_α _= P(-C_α _< N(0,1) < C_α_) = 1- α be a critical threshold *V *is less than -C_α _evidence exists which favors the second model relative to the first. Conversely, if *V *is greater than C_α _then evidence exists which favors the first model relative to the second. Finally, if *V *if less than or equal to C_α _and greater than or equal to -C_α _then weak evidence exists, and we cannot decisively determine which model is favored over the other.

### Statistical Computing

All statistical computation was carried out using SAS version 9.2 (SAS Corporation; Cary, North Carolina). For all regression modeling we used Proc NLMIXED, specifying the likelihood equations, as shown above, and maximizing them directly using numerical methods. Maximization began from various starting points and the final gradient vectors and hessian matrices were investigated to ensure proper convergence of estimated model parameters.

## Results

Descriptive statistics for our sample are presented in Table 1. To account for unequal probabilities of selection and non-response, descriptive statistics are calculated using sampling weights provided by Statistics Canada. The sample size for CCHS cycle 2.1 and 3.1 was 26,693 and 26,660, respectively. Respondents' to CCHS cycle 2.1 were approximately an equal mix of males (50.4%) and females (49.6%). The majority (86.2%) were young-middle aged adults between the ages of 20-64, living in predominantly urban environments (85.9%), with mid-high household incomes (92.2%) and well-educated, receiving at least a high-school diploma (84.1%). In terms of health perceptions, 51.9% of respondents self-reported having no chronic medical conditions and 56.8% rated their self perceived health status as excellent. Most respondents had five or less ADG's (62.3%) indicating a relatively low comorbidity profile in this sample; however, many were ranked as having a high (52.6%) or very-high (15.6%) expected RUB categorization. Finally, most (91.4%) respondents self-reported contact with and easy access to a personal primary care physician in the community. Very similar observations were obtained when we considered CCHS cycle 3.1. A more granular presentation of the socio-demographic and medical characteristics of our sample can be obtained in Table 1.

**Table 1:** Demographic characteristics of CCHS cycle 2

variable	CCHS 2.1		CCHS 3.1	
	N	%	N	%
Female	14086	49.6	14012	49.1
Male	12607	50.4	12648	50.8
Age 20-44	10958	51.6	11549	50.0
Age 45-64	9888	34.6	9627	36.3
Age 65-80	5847	13.8	5484	13.7
Income High	10882	55.1	6739	42.4
Income Medium	10867	37.2	10140	47.4
Income Low	3113	7.7	2948	10.2
Education High	13903	55.4	15177	60.2
Education Medium	7214	28.7	6704	25.9
Education Low	5233	15.9	4655	13.8
No chronic condition	11808	51.9	11890	52.3
One Chronic conditions	7488	27.3	7557	27.2
Two or more chronic conditions	7397	20.8	7213	20.5
Self rated health excellent or very good	14384	56.8	15438	60.5
Self rated health good	8299	31.2	7484	28.2
Self rated health fair or poor	3998	11.9	3721	11.3
ADG 0-5	15888	62.3	16158	63.3
ADG 6-9	7823	28.4	7646	27.3
ADG 10+	2982	9.3	2856	9.4
RUB 0, 1	3433	14.1	3408	14.0
RUB 2	4251	17.7	4382	18.3
RUB 3	13871	52.6	13921	51.8
RUB 4, 5	5138	15.6	4949	15.9
Had regular doctor	24391	91.4	23995	90.5
Didn't have regular doctor	2296	8.6	2656	9.5
Rural	5471	14.1	5409	14.0
Urban	21222	85.9	21251	86.0

**Figure 1:**
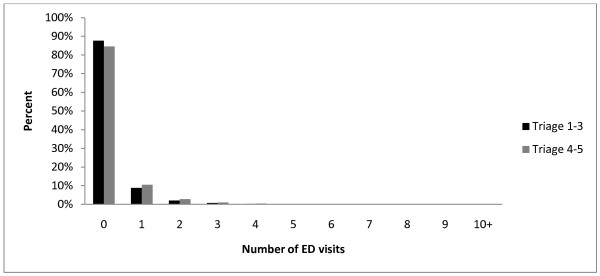
**Histogram of the number of Triage 1-3 (urgent) and Triage 4-5 (less urgent) emergency department (ED) visits**.

Emergency department utilization was determined for each respondent, one year following their respective CCHS 2.1 and 3.1 interview dates. A summary of the respondents utilization patterns was stratified by triage scale, with triage scale rankings 1-3 collapsed into a single category (high severity) and triage scale rankings 4-5 collapsed into a separate category (low severity). Emergency department utilization rates were recorded for both CCHS cycles 2.1 and 3.1 and presented in Table 2. Overall, participants of CCHS 3.1 had higher rates of emergency department utilization compared to participants of CCHS 2.1. The frequency of high severity (triage scale 1-3) emergency department visits ranged from zero (88%) to 28. The frequency of low severity (triage scale 4-5) emergency department visits ranged from zero (85%) to more than 100 visits for a given participant. Overall more than 75% of respondents did not visit the emergency department on any occasion over the 1-year interval following their CCHS interview. Figure 1 displays a histogram representing the distribution of our outcomes, the number of CTAS 1-3 and CTAS 4-5 emergency department visits experienced by cases in our sample. Participants aged 65 and over, having two or more chronic conditions, reporting poor or fair health, having an ADG of ten or higher, and having RUB of four or five had elevated rates of triage scale 1-3 compared to triage 4-5 emergency department visits. Young participants age 20-44 had higher rates of less urgent emergency department visits compared to urgent visits. Being a low income respondent, less educated, having two or more chronic health conditions, reporting fair or poor health, recording 10 or more ADG's, falling into an RUB category of 4 or 5, and living in rural area were also associated with having higher unadjusted rates of emergency department utilization.

**Table 2:** Proportion of persons visiting the emergency department (ED) at least once in a given year and the rate of emergency department visits conditioned on using the emergency department.

	CCHS 2.1				CCHS 3.1			
	Triage 1-3		Triage 4-5		Triage 1 -3		Triage 4-5	
	% ≥ 1 ED visit	Mean (SD)	% ≥ 1 ED visit	Mean (SD)	% ≥ 1 ED visit	Mean (SD)	% ≥ 1 ED visit	Mean (SD)
Female	9.6	1.46(22.69)	11.7	1.57(27.52)	11.8	1.48(17.92)	11.6	1.50(19.52)
Male	10.0	1.51(21.30)	11.3	1.55(25.29)	11.0	1.49(24.51)	11.6	1.50(20.92)
Age 20-44	8.3	1.45(30.23)	12.5	1.49(24.08)	9.9	1.49(27.00)	12.3	1.50(21.49)
Age 45-64	9.7	1.45(17.02)	9.8	1.56(24.29)	11.2	1.40(16.38)	10.1	1.46(19.09)
Age 65-80	15.2	1.62(15.82)	11.9	1.87(33.37)	17.3	1.63(16.88)	12.9	1.60(18.96)
Income High	8.1	1.39(19.60)	9.7	1.43(22.52)	10.5	1.47(28.39)	11.5	1.41(20.12)
Income Medium	11.1	1.52(24.35)	12.8	1.64(25.27)	13.2	1.48(15.60)	12.5	1.60(19.30)
Income Low	15.0	1.63(16.91)	15.3	1.75(23.12)	18.0	1.88(24.83)	15.5	1.94(24.66)
Education High	8.6	1.38(15.73)	9.8	1.54(25.85)	9.9	1.46(22.94)	10.6	1.43(18.98)
Education Medium	10.1	1.57(32.25)	12.2	1.51(23.47)	11.8	1.39(16.59)	12.2	1.50(19.52)
Education Low	13.5	1.61(19.13)	15.9	1.69(30.88)	16.8	1.66(21.57)	14.2	1.74(23.23)
No chronic condition	7.3	1.34(26.11)	10.1	1.48(24.12)	8.5	1.39(20.07)	10.1	1.39(18.72)
One Chronic conditions	9.8	1.54(21.51)	11.5	1.59(26.08)	11.8	1.48(26.00)	12.1	1.43(16.89)
Two or more chronic conditions	16.0	1.60(18.67)	15.0	1.68(29.29)	18.2	1.62(18.05)	14.7	1.78(23.56)
Self rated health excellent	7.5	1.27(12.41)	10.3	1.42(19.85)	8.8	1.37(15.80)	10.0	1.40(17.52)
Self rated health good	10.3	1.47(19.79)	11.4	1.59(28.31)	11.8	1.37(16.98)	13.1	1.47(18.19)
Self rated health fair/poor	19.6	1.89(31.19)	17.4	1.92(34.36)	24.1	1.86(29.94)	16.4	1.92(25.68)
ADG 0-5	6.8	1.35(17.19)	9.5	1.40(18.45)	7.8	1.32(15.25)	10.2	1.36(14.85)
ADG 6-9	12.0	1.43(16.71)	13.2	1.59(27.46)	14.4	1.41(16.20)	12.2	1.60(20.23)
ADG 10+	23.1	1.84(32.50)	19.8	2.03(38.61)	27.1	1.93(31.97)	19.1	1.85(30.32)
RUB 0, 1	4.6	1.20(11.38)	8.0	1.38(18.22)	6.6	1.28(14.38)	9.0	1.30(13.12)
RUB 2	6.5	1.39(21.66)	10.3	1.32(15.15)	6.5	1.25(9.74)	9.5	1.26(11.94)
RUB 3	9.1	1.37(15.53)	10.9	1.53(23.31)	10.9	1.35(15.17)	11.5	1.48(17.67)
RUB 4, 5	20.6	1.74(28.71)	18.1	1.88(36.03)	22.9	1.83(29.22)	16.5	1.82(27.83)
Had regular doctor	10.1	1.50(22.52)	11.5	1.55(26.84)	11.4	1.48(21.47)	11.4	1.47(19.54)
Didn't have regular doctor	7.2	1.31(13.33)	11.6	1.68(23.54)	10.9	1.54(18.51)	13.0	1.78(23.93)
Rural	10.1	1.45(13.81)	17.9	1.70(26.50)	11.4	1.39(13.87)	16.6	1.55(15.47)
Urban	9.8	1.49(23.69)	10.4	1.53(26.53)	11.4	1.50(22.62)	10.8	1.49(21.59)

These statistics are summarized for both low and high severity cases and CCHS cycles.

Rate ratios, odds ratios (where applicable), and 95% confidence intervals for the six count regression models applied to triage scale 1-3 and triage scale 4-5 emergency department visits, from combined CCHS cycles 2.1 and 3.1 data, are presented in Table 3 and Table 4 respectively. Vuong likelihood ratio tests, comparing the 6 count regression models fitted to triage scale 1-3 and triage scale 4-5 are given in Table 5 and Table 6 respectively. Values < -2 indicates that the row model had significantly better fit than the column model and values >2 indicates that column model had significantly better fit than the row model. The results of the Vuong tests suggest that HNB regression is the preferred model among the six candidate regression strategies for modeling triage scale 1-3 emergency department visits. Results of table 3 illustrate that the factors that influence whether a patient does or does not go to the emergency department also influence the intensity of emergency department utilization. Clearly being male, being 20 to 44, having a higher RUB score, having a higher ADG score, being a low income earner, rating health status as good/fair/poor, and having more chronic health conditions are associated with higher rates of emergency department utilization. Having access to a primary care provider or living in rural areas, were not associated with the odds of emergency department utilization, or the rate of emergency department utilization, after controlling for other pertinent factors.

**Table 3:** Regression models for CCHS 2

	Poisson	Negative Binomial	Zero Inflated Poisson		Zero Inflated Negative Binomial		Hurdle Poisson		Hurdle Negative Binomial	
	---	---	Binary	Count	Binary	Count	Binary	Count	Binary	Count
	RR (95% CI)	RR (95% CI)	OR (95% CI)	RR (95%CI)	OR (95% CI)	RR (95% CI)	OR (95% CI)	RR (95% CI)	OR (95% CI)	RR (95% CI)
Male	1.17, (1.12-1.22)**	1.16, (1.10-1.23)**	1.11,(1.01-1.21)*	1.09, (1.02-1.16)*	1.26,(0.96-1.65)	1.13, (1.05-1.22)**	1.17, (1.10-1.23)**	1.09, (1.02-1.16)*	1.17, (1.10-1.23)**	1.09, (0.97-1.22)
Female	---	---	---	---	---	---	---	---	---	---
20-44 years	1.19, (1.12-1.26)**	1.14, (1.05-1.24)**	0.90,(0.80-1.02)	1.25, (1.14-1.37)**	0.66,(0.40-1.09)	1.21, (1.09-1.35)**	1.09, (1.00-1.08)*	1.26, (1.15-1.38)**	1.09, (1.00-1.18)*	1.23, (1.05-1.44)**
45-64 years	0.94, (0.89-0.99)*	0.94, (0.87-1.01)	0.84,(0.75-0.94) **	1.06, (0.97-1.15)	0.76,(0.46-1.26)	0.96, (0.87-1.05)	0.90, (0.83-0.97)**	1.06, (0.97-1.15)	0.90, (0.83-0.97)**	1.06, (0.92-1.22)
> 65 years	---	---	---	---	---	---	---	---	---	---
RUB 2	1.31, (1.17-1.47)**	1.30, (1.14-1.48)**	1.08,(0.81-1.43)	1.23, (0.95-1.58)	1.11,(0.58-2.12)	1.24, (0.93-1.66)	1.27, (1.11-1.45)**	1.23, (0.95-1.58)	1.27, (1.11-1.45)**	1.24, (0.89-1.72)
RUB 3	1.65, (1.49-1.83)**	1.62, (1.44-1.82)**	1.15,(0.89-1.48)	1.46, (1.16-1.83)**	1.25,(0.70-2.24)	1.45, (1.12-1.88)**	1.54, (1.36-1.74)**	1.46, (1.17-1.83)**	1.54, (1.36-1.74)**	1.50, (1.12-2.01)**
RUB 4-5	2.54, (2.25-2.85)**	2.49, (2.16-2.86)**	1.32,(1.00-1.75)	2.04, (1.60-2.59)**	2.04,(0.84-4.98)	2.13, (1.62-2.81)**	2.23, (1.93-2.58)**	2.05, (1.61-2.60)**	2.23, (1.93-2.58)**	2.23, (1.61-3.09)**
RUB 1	---	---	---	---	---	---	---	---	---	---
ADG 6-9	1.45, (1.37-1.54)**	1.45, (1.35-1.56)**	1.52,(1.34-1.73) **	1.06, (0.96-1.17)	4.44,(2.16-9.13) **	1.08, (0.94-1.23)	1.50, (1.39-1.62)**	1.06, (0.96-1.18)	1.50, (1.39-1.62)**	1.07, (0.92-1.25)
ADG 10-32	2.22, (2.05-2.39)**	2.18, (1.96-2.43)**	1.82,(1.55-2.15) **	1.43, (1.27-1.62)**	-	1.51, (1.29-1.78)**	2.17, (1.95-2.41)**	1.44, (1.28-1.63)**	2.17, (1.95-2.41)**	1.56, (1.28-1.91)**
ADG 0-5	---	---	---	---	---	---	---	---	---	---
Income Low	1.46, (1.37-1.56)**	1.46, (1.34-1.59)**	1.11,(0.98-1.27)	1.34, (1.22-1.48)**	0.89,(0.59-1.34)	1.45, (1.29-1.63)**	1.37, (1.25-1.49)**	1.35, (1.22-1.49)**	1.37, (1.25-1.49)**	1.47, (1.25-1.74)**
Income Med.	1.16, (1.10-1.22)**	1.19, (1.11-1.27)**	1.04,(0.94-1.16)	1.14, (1.05-1.24)**	1.47,(1.05-2.05) *	1.09, (1.00-1.20)*	1.14, (1.07-1.22)**	1.14, (1.05-1.24)**	1.14, (1.07-1.22)**	1.19, (1.04-1.35)*
Income High	---	---	---	---	---	---	---	---	---	---
Educ. Low	1.17, (1.11-1.23)**	1.20, (1.11-1.29)**	1.23,(1.10-1.38) **	1.02, (0.94-1.10)	1.28,(0.85-1.93)	1.15, (1.04-1.26)**	1.21, (1.12-1.30)**	1.01, (0.93-1.10)	1.21, (1.12-1.30)**	1.08, (0.94-1.24)
Educ. Med.	1.04, (0.99-1.09)	1.06, (0.99-1.13)	1.09,(0.98-1.21)	0.98, (0.90-1.07)	1.16,(0.84-1.60)	1.02, (0.93-1.12)	1.06, (0.99-1.14)	0.98, (0.90-1.06)	1.06, (0.99-1.14)	1.00, (0.87-1.14)
Educ. High	---	---	---	---	---	---	---	---	---	---
SRH Poor or Fair	1.96, (1.85-2.08)**	1.91, (1.76-2.08)**	1.36,(1.19-1.55) **	1.57, (1.43-1.73)**	0.91,(0.58-1.43)	1.99, (1.80-2.20)**	1.82, (1.68-1.97)**	1.58, (1.44-1.74)**	1.82, (1.68-1.97)**	1.73, (1.48-2.01)**
SRH Good	1.21, (1.17-1.30)**	1.21, (1.13-1.29)**	1.07,(0.95-1.20)	1.16, (1.05-1.27)**	0.72,(0.53-0.93) *	1.30, (1.18-1.42)**	1.19, (1.12-1.28)**	1.16, (1.06-1.27)**	1.19, (1.12-1.28)**	1.18, (1.02-1.35)*
SRH Excellent or Very Good	---	---	---	---	---	---	---	---	---	---
Access Doctor	0.92, (0.85-1.00)*	0.92, (0.83-1.02)**	0.88,(0.75-1.05)	1.01, (0.88-1.15)	0.77,(0.48-1.25)	0.97, (0.83-1.13)	0.90, (0.81-1.00)	1.01, (0.89-1.15)	0.90, (0.81-1.00)	0.99, (0.88-1.23)
No Doctor	---	---	---	---	---	---	---	---	---	---
1 Chronic Condition	1.13, (1.07-1.20)**	1.13, (1.05-1.22)**	0.99,(0.88-1.12)	1.13, (1.02-1.24)*	1.06,(0.76-1.48)	1.10, (0.99-1.22)	1.09, (1.01-1.17)*	1.13, (1.03-1.25)*	1.09, (1.01-1.17)*	1.19, (1.03-1.39)*
>2 Chronic Conditions	1.19, (1.11-1.26)**	1.21, (1.12-1.31)**	1.24,(1.09-1.42) **	1.02, (0.92-1.13)	1.80,(1.10-2.95) *	1.10, (0.98-1.23)	1.22, (1.13-1.33)**	1.02, (0.92-1.13)	1.22, (1.13-1.33)**	1.07, (0.91-1.26)
No Chronic Condition	---	---	---	---	---	---	---	---	---	---
Rural	0.97, (0.92-1.02)	0.99, (0.93-1.07)	1.00,(0.89-1.12)	0.99, (0.91-1.08)	1.73,(1.08-2.75)*	0.90, (0.82-0.99)*	0.99, (0.92-1.06)	0.98, (0.90-1.07)	0.99, (0.92-1.06)	0.98, (0.85-1.12)
Urban	---	---	---	---	---	---	---	---	---	---

*Statistically significant at 0.05 level

**Statistically significant at 0.01 level

**Table 4:** Regression models for CCHS 2.1 and 3.1 combined. Triage scale 4-5.

	Poisson	Negative Binomial	Zero Inflated Poisson		Zero Inflated Negative Binomial		Hurdle Poisson		Hurdle Negative Binomial	
	---	---	Binary	Count	Binary	Count	Binary	Count	Binary	Count
	RR (95% CI)	RR (95% CI)	OR (95% CI)	RR (95%CI)	OR (95% CI)	RR (95% CI)	OR (95% CI)	RR (95% CI)	OR (95% CI)	RR (95% CI)
Male	1.10, (1.06-1.14) **	1.11, (1.05-1.17)**	0.94,(0.88-1.01)	1.16, (1.10-1.23)**	0.23,(0.07-0.81) *	1.15, (1.08-1.21)**	1.05, (1.00-1.11)	1.15, (1.09-1.21)**	1.05, (1.00-1.11)	1.20, (1.09-1.33)**
Female	---	---	---	---	---	---	---	---	---	---
20-44 years	1.88, (1.79-1.98)**	1.82, (1.68-1.97)**	1.52,(1.37-1.68)**	1.38, (1.29-1.49)**	1.78,(0.43-7.35)	1.78, (1.64-1.93)**	1.80, (1.67-1.95)**	1.38, (1.29-1.49)**	1.80, (1.67-1.95)**	1.42, (1.23-1.63)**
45-64 years	1.18, (1.12-1.24)**	1.16, (1.07-1.25)**	1.11,(1.01-1.22) *	1.10, (1.02-1.18)**	0.45,(0.11-1.81)	1.18, (1.09-1.27)**	1.16, (1.08-1.25)**	1.10, (1.02-1.17)**	1.16, (1.08-1.25)**	1.08, (0.95-1.24)
> 65 years	---	---	---	---	---	---	---	---	---	---
RUB 2	1.25, (1.15-1.36)**	1.22, (1.10-1.36)**	1.16,(0.98-1.38)	1.09, (0.94-1.26)	3.16,(0.74-13.44)	1.16, (1.02-1.31)*	1.21, (1.09-1.34)**	1.11, (0.96-1.29)	1.21, (1.09-1.34)**	1.13, (0.91-1.41)
RUB 3	1.61, (1.50-1.74)**	1.55, (1.40-1.71)**	1.19,(1.02-1.39) *	1.37, (1.20-1.55)**	3.98,(0.94-16.84)	1.47, (1.32-1.65)**	1.46, (1.33-1.61)**	1.41, (1.24-1.60)**	1.46, (1.33-1.61)**	1.48, (1.22-1.80)**
RUB 4-5	1.79, (1.63-1.95)**	1.69, (1.49-1.91)**	1.28,(1.07-1.54)**	1.42, (1.23-1.65)**	-	1.53, (1.33-1.75)**	1.59, (1.41-1.80)**	1.48, (1.28-1.71)**	1.59, (1.41-1.80)**	1.56, (1.23-1.98)**
RUB 1	---	---	---	---	---	---	---	---	---	---
ADG 6-9	1.39, (1.33-1.46)**	1.37, (1.28-1.47)**	1.09,(0.99-1.20)	1.30, (1.21-1.39)**	0.11,(0.02-0.55) **	1.45, (1.35-1.56)**	1.30, (1.21-1.38)**	1.30, (1.21-1.40)**	1.29, (1.21-1.38)**	1.39, (1.23-1.57)**
ADG 10-32	2.19, (2.04-2.34)**	2.16, (1.94-2.41)**	1.27,(1.11-1.45)**	1.84, (1.67-2.02)**	0.07,(0.01-0.48) **	2.29, (2.04-2.55)**	1.80, (1.62-2.00)**	1.84, (1.67-2.02)**	1.80, (1.62-1.99)**	2.21, (1.83-2.65)**
ADG 0-5	---	---	---	---	---	---	---	---	---	---
Income Low	1.62, (1.53-1.71)**	1.53, (1.40-1.66)**	1.18,(1.06-1.32) *	1.40, (1.29-1.51)**	0.22,(0.07-0.73) *	1.58, (1.45-1.73)**	1.45, (1.33-1.57)**	1.41, (1.31-1.53)**	1.45, (1.33-1.57)**	1.47, (1.26-1.70)**
Income Med.	1.32, (1.27-1.38)**	1.30, (1.23-1.38)**	1.07,(0.98-1.16)	1.25, (1.18-1.33)**	0.63,(0.26-1.50)	1.32, (1.24-1.41)**	1.24, (1.17-1.31)**	1.26, (1.18-1.34)**	1.24, (1.17-1.31)**	1.31, (1.18-1.46)**
Income High	---	---	---	---	---	---	---	---	---	---
Educ. Low	1.27, (1.21-1.33)**	1.26, (1.17-1.35)**	1.21,(1.10-1.32) *	1.12, (1.05-1.19)**	-	1.20, (1.11-1.29)**	1.27, (1.19-1.36)**	1.11, (1.04-1.18)**	1.27, (1.19-1.36)**	1.12, (0.99-1.27)
Educ. Med.	1.08, (1.03-1.12)**	1.08, (1.02-1.15)*	1.08,(0.99-1.17)	1.03, (0.97-1.10)	6.40,(1.39-29.47) *	1.04, (0.97-1.11)	1.09, (1.03-1.16)**	1.02, (0.96-1.09)	1.09, (1.03-1.16)**	1.02, (0.91-1.14)
Educ. High	---	---	---	---	---	---	---	---	---	---
SRH Poor	1.50, (1.42-1.58)**	1.51, (1.39-1.64)**	1.07,(0.97-1.19)	1.41, (1.31-1.52)**	-	1.49, (1.37-1.61)**	1.32, (1.22-1.43)**	1.42, (1.32-1.53)**	1.32, (1.22-1.43)**	1.62, (1.41-1.86)**
SRH Good	1.16, (1.11-1.20)**	1.14, (1.07-1.22)**	1.05,(0.97-1.15)	1.10, (1.03-1.17)**	0.62,(0.31-1.27)	1.16, (1.08-1.23)**	1.12, (1.06-1.19)**	1.11, (1.04-1.18)**	1.12, (1.06-1.19)**	1.14, (1.02-1.27)*
SRH Excellent	---	---	---	---	---	---	---	---	---	---
Access Doctor	0.58, (0.55-0.61)**	0.61, (0.56-0.67)**	0.86,(0.78-0.96) *	0.69, (0.64-0.74)**	3.49,(1.65-7.38) **	0.58, (0.53-0.64)**	0.69, (0.63-0.75)**	0.67, (0.62-0.72)**	0.69, (0.63-0.75)**	0.57, (0.50-0.66)**
No Doctor	---	---	---	---	---	---	---	---	---	---
1 Chronic Conditions	1.10, (1.05-1.15)**	1.09, (1.02-1.17)*	1.18,(1.08-1.30) *	0.97, (0.91-1.04)	1.68,(0.80-3.52)	1.07, (1.00-1.15)	1.14, (1.07-1.22)**	0.97, (0.90-1.04)	1.14, (1.07-1.22)**	0.92, (0.82-1.04)
>2 Chronic Condition	1.25, (1.19-1.32)**	1.26, (1.16-1.36)**	1.25,(1.13-1.38)**	1.06, (0.99-1.14)	-	1.19, (1.10-1.29)**	1.27, (1.18-1.37)**	1.06, (0.99-1.14)	1.27, (1.18-1.37)**	1.08, (0.94-1.24)
No Chronic Condition	---	---	---	---	---	---	---	---	---	---
Rural	1.59, (1.53-1.65)**	1.61, (1.52-1.71)**	1.56,(1.44-1.69)**	1.16, (1.10-1.23)**	-	1.55, (1.45-1.65)**	1.64, (1.55-1.74)**	1.16, (1.10-1.23)**	1.64, (1.55-1.74)**	1.23, (1.11-1.37)**
Urban	---	---	---	---	---	---	---	---	---	---

*Statistically significant at 0.05 level

**Statistically significant at 0.01 level

**Table 5:** Vuong Likelihood-ratio statistics comparing non-nested models. Triage scale 1-3

	Poisson	NB	ZIP	ZINB	HP	HNB
Poisson	---					
NB	-3.66	---				
ZIP	-4.76	-4.97	---			
ZINB	-4.27	-4.36	-3.82	---		
HP	-4.77	-5.00	-3.43	3.81	---	
HNB	-4.25	-4.32	-3.81	-2.69	-3.80	---

*Values < -2 indicates the row model had significantly better fit than the column model.

*Values >2 indicates that column model had significantly better fit than the row model.

**Table 6:** Vuong Likelihood-ratio statistics comparing non-nested models.

	Poisson	NB	ZIP	ZINB	HP	HNB
Poisson	---					
NB	-3.82	---				
ZIP	-5.30	-6.25	---			
ZINB	-4.58	-4.75	-4.06	---		
HP	-5.36	-6.34	-3.92	4.02	---	
HNB	-4.58	-4.74	-4.07	-2.18	-4.03	---

Triage scale 4-5.

*Values < -2 indicates the row model had significantly better fit than the column model.

*Values >2 indicates that column model had significantly better fit than the row model.

Similarly, when the Vuong test is applied to the combined CCHS cycle 2.1 and 3.1 dataset, stratified by low severity (triage scale 4-5) emergency department visits, the results suggest that the HNB model is a good fit for these data (Table 6). Results of Table 4 showed that being less than 65 years of age, having higher RUB and ADG scores, being a low income earner or a less educated person, not having excellent self-perceived health status, not having regular primary care provider, having more chronic conditions, and living in rural areas are factors that increase the odds of visiting the emergency department with triage scale 4-5 conditions at least once during the one year period of observation following the CCHS interview. Of interest, the probability of going to emergency department was not influenced by gender. However among those who utilized emergency department with triage 4 and 5, males had higher rate of utilization. Those participants who had access to family physician had a lower odds of using the emergency department (OR = 0.69, 95% CI, 0.63-0.75, P < 0.01) and also a lower rate of emergency department utilization (RR = 0.57; 95% CI, 0.50 - 0.66, p < 0.01). Respondents who lived in rural areas also had a higher probability of going to emergency department (OR = 1.64; 95% CI, 1.55 - 1.74, p < 0.01) and a higher rate of emergency department visits (RR = 1.23; 95% CI, 1.11 - 1.37, p < 0.01).

## Discussion

Poisson regression is a commonly employed method for analyzing count data. Our results illustrate that the Poisson regression model is a candidate model for analyzing the number of emergency department visits observed in the CCHS 2.1 and 3.1 datasets; however, alternative methodologies exist which may yield better fits to the observed data. Extra variation in the count data can be handled by extensions to the familiar Poisson model or by using a NB regression approach. Health utilization data, such as the number of emergency department visits made by an individual during a fixed window of follow-up time, are typically characterized by a large proportion of zeroes, representing those individuals who exhibit zero demand for the service during the study interval. Further, some individuals exhibit large demand for emergency department services, resulting in an empirical distribution of counts with a long right tail and extra-Poisson variation. Modified Poisson and NB regression models are able to deal with both extra variation (overdispersion) and the excess of zeros which are typically observed in medical utilization data. The HNB model is an extension of the NB model (which itself, is an extension of the Poisson model) and is a natural choice for modeling data that exhibit both extra variation and excess of zeros, especially when zeros are structural. Although the NB regression model fits these data well, and has fewer estimated parameters than the HNB model, we tend to favor the slightly more complex hurdle model. The theoretical framework of the HNB model is an ideal choice for modeling medical utilization data as it allows researchers to simultaneously interpret the factors which influence the odds of using the medical service and the rate/intensity at which utilization occurs in those who do exhibit positive demand for the service.

Our results demonstrate the suitability of both the NB model and the HNB model for analyzing emergency department demand in the CCHS cycle 2.1 and 3.1 datasets. As an aside the ZINB model also fit these data well; however, the zeroes in this model are a mix from the Bernoulli component of the model and the count component of the model, and hence interpretation is not as simple. The Vuong test, which is designed for comparing non-nested regression models, suggests the HNB model is the most appropriate approach to modeling emergency department demand in this study.

The impact of covariates on the odds of visiting the emergency department for a less severe visit (triage scale 4-5) versus a more severe visits (triage scale 1-3) are quite different. For example, gender is not associated with the likelihood of emergency department utilization in the analysis characterized by less severe visits. However, male gender is statistically significantly associated with increased odds of at least one emergency department visit in the analysis stratified by more severe cases. This result indicates the importance of stratifying our analyses according to the severity of the triage scale, as the factors influencing the emergency department utilization may vary as a function of the severity of a cases initial presentation.

The impact of access to a primary care physician on emergency department utilization rates is an interesting finding in our analysis. Once again, the impact of this covariate differs according to the severity of presentation. For more severe cases (triage scale 1-3), having access to a family doctor did not influence the odds of emergency department utilization, nor did it impact the rate of utilization in those who demonstrated positive demand for the service over the study interval. For less severe emergency department visits (triage scale 4-5) we estimate that having access to a primary care provider significantly reduces the likelihood (OR = 0.69) of a visit. Further, given that a visit occurs, the rate of utilization is also significantly lower in those with access to a primary care provider (RR = 0.57). From a policy perspective, this finding suggests that having access to a primary care provider has the opportunity to reduce more than 40 percent of less urgent emergency department visits. Hence, strategies to increase the supply/access to primary health care professionals may result in reduced demand for emergency department services and fewer issues related to crowding, wait times and variable quality of care in Ontario's emergency departments.

To our knowledge this study is a unique population based Canadian study, which links a large national survey to provincial health utilization databases to assess the impact of individual level characteristics on the emergency department demand. Our sample size is large and outcome measures are complete. Results of this study are based on regression models that are theoretically appropriate and statistically had the best fit compared to other potential models which were investigated. Some of the findings of this study have important policy implications and if adopted may result in reducing the number of less urgent emergency department visits that are occurring in Ontario.

One limitation of our study is that we did not examine the impact of contextual factors, such as: accessibility to nearby walk in clinics, the number of primary care providers in a respondents' census tract or postal code region or the distance to nearest emergency department at the area level. Nor did we stratify our analyses according to other pertinent factors, such as: the day of the week (weekday versus weekend) or the time of the day. An advanced multi-level modeling framework can be extended to the HNB regression model fit to these data to assess the impact of contextual factors on the likelihood and intensity of emergency department visits when the impact of individual level characteristics are adjusted for. Similar methodological approaches can be adopted for stratified analyses.

## Conclusions

Demand for emergency department services can be appropriately modeled using simple extensions to count based regression models, such as the HNB model. This model simultaneously accounts for excess zeroes, a skewed empirical distribution (extra-variation) and unobserved heterogeneity that is common in medical demand data. Additionally, the two component interpretation of the hurdle models makes them ideal for understanding factors which affect those who experience no demand for emergency department services versus those persons that experience positive demand for emergency department services.

This analysis also revealed that the factors which influence the likelihood and intensity of emergency department services vary according to the severity of initial presentation. Some important factors that differed between the two stratified analyses were access to a primary care physician and urban-versus-rural residence. While access to a primary care physician was an irrelevant factor on both the odds and intensity of emergency department utilization in high severity cases, this factor was a statistically significant predictor of the likelihood and rate of emergency department services in low severity cases. Our findings suggest that access to a primary care physician could reduce the odds of a low severity emergency department visit by approximately 31% and further reduce the rate of low severity emergency department visits by approximately 43%. This suggests that re-structuring health care services in Ontario, such that access to primary care physicians is enhanced, may result in a reduced number of low severity cases presenting in the emergency department.

## Competing interests

The authors declare that they have no competing interests.

## Authors' contributions

RM performed the analysis and interpreted the results. RM and CM drafted the paper. MA and BZ cut the data. RM and RHG conceptualized the research. All authors read and approved the final manuscript.

## Pre-publication history

The pre-publication history for this paper can be accessed here:

http://www.biomedcentral.com/1471-227X/11/13/prepub
